# A new scale to assess technostress levels in an Italian banking context: the Work-Related Technostress Questionnaire

**DOI:** 10.3389/fpsyg.2023.1253960

**Published:** 2023-09-01

**Authors:** Desirée Estela Porcari, Emiliano Ricciardi, Maria Donata Orfei

**Affiliations:** Molecular Mind Laboratory (MoMiLab), IMT School for Advanced Studies Lucca, Lucca, Italy

**Keywords:** technostress, workplace, information and communication technology, questionnaire, validation

## Abstract

**Introduction:**

Technostress (TS) represents a multidimensional phenomenon closely related to the pervasive use of information and communication technologies. This study aimed to validate a new psychometric tool for assessing TS in an Italian banking context, the Work-Related Technostress – Questionnaire (WRT-Q). Secondly, we analyzed the role of gender and age in modulating TS manifestations.

**Methods:**

A sample of 2,586 bank employees (51% females; age: 47.26  ±  8.6) underwent an online survey. Reliability, exploratory factor analysis (EFA), confirmatory factor analysis (CFA), ANCOVA, independent sample *t*-test, and correlation analyses were performed.

**Results:**

The WRT-Q consisted of 17 items and a four-factor structure, supported by the following CFA indices: Comparative Fit Index (CFI)  =  0.985; Incremental Fit Index (IFI)  =  0.985; Goodness of Fit (GFI)  =  0.988; Root Mean Squared Error of Approximation (RMSEA)  =  0.071; and SRMR  =  0.062. A significant difference in TS levels between age classes emerged (*p* < 0.001) with higher levels in the over 55-year-old subgroup, while no statistically significant difference emerged for gender. Moreover, the whole sample found a significant positive association between age and TS (*p* < 0.001).

**Discussion:**

The WRT-Q is a new instrument to measure TS in the workplace, it can contribute to highlighting adverse outcomes in individuals due to a dysfunctional interaction with ICT.

## 1. Introduction

Information and Communication Technology (ICT) refers to devices, networking components, applications, and systems that allow people and businesses to interact in the digital world (Steinmueller, 2000; Day et al., 2010).

Within the industrial age, technologies were mainly designed to reduce physical efforts. In the information age, ICTs are involved primarily in saving workers’ cognitive and social efforts (Wang et al., 2020). However, the massive use of ICT increased job demands and expectations, negatively impacting users’ emotions, well-being, and performance (Bessière et al., 2006; Ragu-Nathan et al., 2008; Zimmerman et al., 2014). Despite the indisputable advantages ICT brings, a large body of research has shown the negative consequences of intensive work-related ICT use, such as work–family conflict, emotional exhaustion, poor sleep quality, and worse performances (Boswell and Olson-Buchanan, 2007; Chen and Karahanna, 2014; Butts et al., 2015; Ferguson et al., 2016; Piszczek, 2017; Borle et al., 2021). Specifically, the term *technostress* (TS) was coined to indicate the inability to interact with ICT in a healthy manner (Brod, 1984). Years later, the TS definition was extended to any negative effect on behavioral, mental, and physical well-being caused by technology’s direct or indirect use (Weil and Rosen, 1997). More recently, TS has been defined as a state of psychophysiological stimulation caused by the use of ICT for work, generally associated with increasing work overload and a decrease in personal time (Lei and Ngai, 2014). In this perspective, TS loses any pathological reference but instead describes a consequence of technology use on individuals’ well-being, the so-called “dark side” of ICT use for people and organizations (Salanova et al., 2013; Bondanini et al., 2020).

### 1.1. Emotional and physiological adverse effects of TS

A dysfunctional ICT use can exacerbate work-related stress, defined as the physical and emotional response occurring when job demands do not match a worker’s capabilities, resources, and needs (National Institute for Occupational Safety and Health [NIOSH], 1999), increasing cognitive overload, role ambiguity, and job insecurity (Grant et al., 2013). Two psychological experiences characterize dysfunctional ICT interaction: techno-strain and techno-addiction (Salanova et al., 2013). Techno-strain is the perceived stress experience resulting from the use of ICT. It is characterized by a combination of high levels of anxiety (e.g., fear, apprehension, and agitation), fatigue (e.g., lower levels of psychological activation), skepticism (e.g., cognitive distancing and indifference), and inefficacy (e.g., sense of inability and self-esteem) related to the use of ICT. At the same time, techno-addiction consists of an uncontrollable compulsion to use ICT for long periods in an excessive way. Techno-addiction stems from an internal need to interact with ICT, which leads to a compulsive use of ICT even in the absence of objective work demands and to ruminations. It is strictly related to workaholic and telepressure phenomena, such as the fear of losing job requests and important messages, even if these occur outside of office time, to be deemed inefficient and not be able to carry out assigned activities. Techno-addiction and techno-strain are strictly related since techno-addicted users are also anxiously compelled to use ICT, thus resulting in intense psychophysical fatigue experience (Salanova et al., 2013). The detrimental impact of TS on workers implies experiencing burnout, depression, anxiety, and perceived social pressure to be constantly available or connected and to prove capabilities at multitasking (Reinecke et al., 2017), as well as cognitive symptoms, such as poor concentration, and memory disturbances (Arnetz and Wiholm, 1997). TS is characterized by the activation of the hypothalamus-hypophysis-adrenal gland axis that causes an increase in blood cortisol levels (Riedl, 2012). In addition, a study showed that workers subjected to the techno-stressor condition had a higher level of heart rate variability and a higher level of salivary stress enzyme α-amylase (sAA) than the control group (Tams et al., 2014).

### 1.2. Theoretical models of TS

An approach frequently used in the literature is the operationalization of TS utilizing the measurement of TS creators (TSCs), i.e., organizational stressors associated with the inefficient use of ICT that affect an individual’s well-being and engender TS. In this context, different theoretical models have been developed to describe TS, such as the five techno-stressor framework (Tarafdar et al., 2007), the transactional model (Ragu-Nathan et al., 2008), the Person-Environment model (Ayyagari et al., 2011), and the model based on RED (resources-demands-experiences) framework (Salanova et al., 2013).

The five-stressor model (Tarafdar et al., 2007) and the transactional model (Ragu-Nathan et al., 2008) focus on TSCs. Specifically, the authors proposed that TS can be measured considering five (techno-overload, techno-invasion, techno-complexity, techno-insecurity, and techno-uncertainty) or three (stressors, situational factors, and strain) detrimental effects reported by users, respectively. The five-stressor model considers TSCs as a single construct with a very nature that leads to the same outcomes regardless of the relationship between people and the environment. By contrast, the transactional model considers TSCs dynamic since an individual’s perception of TS depends on the situational context (Srivastava et al., 2015; Saidy et al., 2022). Considering the bidirectional and mutually reciprocal relationship between people and the environment and the continually evolving nature of ICT, we believe that the latest model contributes to a comprehensive vision of the TS phenomenon. Differently, Ayyagari et al. (2011) focused on ICT characteristics, like usability, intrusiveness, and dynamism, proposed to be related to five stressors (work overload, role ambiguity, invasion of privacy, work-home conflicts, and job insecurity). Lastly, the model proposed by Salanova et al. (2013) postulates that the TS experienced at work is determined by the unbalance between job demands and personal resources to cope with them. Thus, as a general consideration, ICTs are not stressful *per se*, but the job demands, situational factors, availability of resources, and personal characteristics can determine stressful interaction and TS.

### 1.3. Assessment of TS

The five-factors model mainly contributed to studying TS manifestations in work or other life contexts. The questionnaire developed by Tarafdar et al. (2010) has been used in several cross-sectional studies (La Torre et al., 2018, 2020). Other instruments were developed based on this model (Ragu-Nathan et al., 2008; Nimrod, 2018; Fischer et al., 2021). The Technostress Creators Inventory (Ragu-Nathan et al., 2008) consists of 23 items, divided into five subscales (techno-overload, techno-invasion, techno-complexity, techno-insecurity, and techno-uncertainty). The validation process showed data collection from five organizations and a sample size of 608 respondents. However, the study was characterized by selection biases like firm-specific samples, selection of organizations based on researchers’ contacts, and self-selection of respondents. Although this inventory is widely used for different purposes, it is considered out-of-date (Fischer et al., 2019). Specifically, Fischer and colleagues (Fischer et al., 2021) included in the inventory more recent constructs like techno-unreliability (malfunctions and unexpected system behaviors), IT-based monitoring (workers’ behaviors can be tracked by technology), and cyberbullying (the use of ICT for negative behaviors like offensive comments and insults). The authors found that techno-insecurity was the least prevalent stressor category, while techno-unreliability was the most pervasive stressor. However, the validation of this new inventory version was characterized by a small sample and the same selection biases as the previous study (Ragu-Nathan et al., 2008), resulting in not being generalizable to a broader population.

Nimrod (2018) developed an instrument inquiring TS into five domains: overload (having to cope with more tasks and performing them rapidly), invasion (blurred boundaries between public and personal context), complexity (constant change of ICT makes them challenging to use), privacy (threat for personal information) and inclusion (low self-esteem compared to younger users and continuous effort to be included in the contemporary technological environment). However, the validation was performed on an older population (from 60 years old), thus limiting the generalizability of results. Moreover, it focuses on work and not-work contexts, thus possibly including heterogeneous behaviors and strains. Differently, the Digital Stressors Scale (Fischer et al., 2021) focuses on TS in the workplace context and comprises 50 items. Its ten subscales reflect specific stressors: the complexity of technology, conflicts between work and private life, job insecurity, the privacy of technology use, overload, technology safety, pressure from the social environment, lack of technical support, lack of technology usefulness, and technology unreliability. Although allowing an extensive assessment of TS and being validated on a large sample of the US-employed population (*N* = 1,998), this questionnaire requires a long time to be filled out and does not include any psychophysical manifestations of TS (e.g., irritability, anxiety, demotivation, fatigue, loss of concentration, insomnia, and migraine).

In conclusion, the existing tools to assess TS, in particular in the Italian language, may not be entirely satisfactory, manageable, and flexible due to dated theoretical models, selection biases, specific contexts of application, or excessive length. In particular, if aimed at organization contexts, the size of a questionnaire is not a secondary aspect: short questionnaires should be preferred in the workplace context to be included effortlessly in occupational health surveillance routine In fact, timely information gained in the workplace are necessary for organizations to plan, implement and evaluate preventive interventions (Soleo et al., 2006). Especially in large organizations, these articulated visits may require longer time and tools have to be easy and rapid to administer, cost-effective and easy to interpret (Serra et al., 2007). Moreover, the information provided by the tool have to be clear and not ambiguous as well as have to address the core issues of the phenomenon of interest to orientate efficiently the physician in his/her screening activity. As discussed in the other section of this paper, it is established that TS may affect psychological and physical well-being; however, to the best of our knowledge, psychophysical manifestations of TS gain low attention in the previous assessment tools.

### 1.4. Organizational safety culture and aims of the study

The organizational safety culture has been established within business companies in recent years. It emphasizes workers’ safety by each group member and at every level of the organization, considering that employees’ well-being directly affects performance and profit (Brivio et al., 2018). Safety culture means not only regulations and transmission of information but also health surveillance procedures. TS may be an expression of a lack of safety culture and any intervention to recognize and prevent TS may positively impact employees’ performance and well-being (La Torre et al., 2020; Salazar-Concha et al., 2021). Thus, an efficient assessment of technostress may greatly contribute to improving the quality of life in the workplace.

The present study aims to introduce the Work-Related Technostress Questionnaire (WRT-Q), a short and easy-to-use questionnaire, to assess TS specifically in the Italian banking context and focus on the main indicators of the phenomenon, regardless of the kind of ICTs used. The WRT-Q is supposed to overcome some of the above-mentioned gaps and limitations of previous assessment tools, namely excessive length, low flexibility in routinary health surveillance procedures, applicability to different employment job roles and various ICT devices and tools. In the perspective of the present study, as a general theoretical framework, we refer to the transactional model and more specifically suggest defining TS as a detrimental psychophysical reaction due to an unhealthy relationship with ICT in the workplace, jeopardizing the quality of work life, determining emotional and cognitive distress, and triggering signs of psychophysical discomfort.

As a secondary goal, we analyzed the role of socio-demographic variables, namely gender, and age, in modulating TS manifestations. Regarding work-related TS, age, and gender are antecedents, i.e., factors that may provoke more frequent TS occurrence or even amplify the level of TS (La Torre et al., 2018). Empirical evidence on the relation between these variables and TS is mixed. Some studies showed a positive relationship between age and TS levels (Tarafdar et al., 2011; Riedl et al., 2012; Brivio et al., 2018), mostly due to greater difficulty handling ICT and to resistance to novelty. However, a minority of studies stressing that younger subjects may show higher levels of TS should be considered (Ragu-Nathan et al., 2008). Regarding gender results, in literature there are less reliable data. Some studies showed that men develop a higher level of TS than women (Ragu-Nathan et al., 2008; Tarafdar et al., 2011; Riedl et al., 2012), while other studies showed opposite evidence (Marchiori et al., 2019; La Torre et al., 2020). In the present study, we expected: (1) women showing higher levels of TS than men, as previous studies showed a greater tendency to workaholism and work-life conflict in the female gender, two aspects which may be enhanced by an invasive use of ICT (Orfei et al., 2022); and (2) older workers showing higher levels of TS than younger subjects due to greater difficulties to adapt to new technologies and innovation (Day et al., 2010; O’Driscoll et al., 2010).

## 2. Materials and methods

### 2.1. Study design

A cross-sectional web-based study was performed. The survey included a questionnaire on TS and questions about socio-demographic data. All participants were provided with a detailed description of the experimental procedures and required consent before participating in the study. The survey was anonymous since each participant was assigned an alphanumeric code. We collected data from April 19th to May 10th, 2021, and the survey was evenly distributed across the national territory. The study was conducted following the ethical standards laid down in the 1964 Declaration of Helsinki and under a protocol approved by the Joint Ethical Committee for Research of Scuola Normale Superiore, Scuola Superiore Sant’Anna, and IMT School for Advanced Studies Lucca (protocol n. 04/2021).

### 2.2. Participants

A panel of 8,306 employees of a large Italian banking group whose daily work activities imply ICT use was invited to participate in an online survey. Inclusion criteria were: (a) age higher or equal to 18 years old and (b) Italian mother tongue or high-level knowledge of the Italian language; and (c) use of ICTs (personal computer, tablet, smartphone, software, and messaging and video calling tools) during one’s everyday work activity. The subjects covered different roles in the bank (i.e., responsible, not responsible and operational coordinator of activities) and they were part of different business units (BU), i.e., government compliance area, legacy, operations, safety and protection department, organization, people management and human resources transformation, learning academy, corporate and, management systems, and finance. Moreover, the participants were allocated to the whole national territory. From the initial panel 2,586 participants (51% females; age: 47.26 ± 8.6) filled in the survey.

We considered age both as a continuous variable and a categorical variable. For the categorical approach, according to the literature, we categorized age into three groups (under 35 years old, 36–54 years old, and over 55 years old) (De Rosa et al., 2014; ISTAT, 2020; OECD, 2021).

### 2.3. Measure development and item generation

Before developing the questionnaire, according to our theoretical framework of reference and to the above mentioned definition of TS, we identified three core dimensions to investigate by our questionnaire: (a) causal attribution, i.e., attributing the leading cause of one’s worsening performance and cognitive well-being to the use of technology, (b) stress and emotional reactions, i.e., feelings like burnout, lack of work-motivation, irritability, and (c) loss of control, i.e., techno-addiction and sense of invasion. The first draft of the questionnaire consisted of 35 items. Two of the authors (Emiliano Ricciardi, and Maria Donata Orfei) assessed content validity of each item on a 4-point scale item as follows: 1 (irrelevant), 2 (equivocal or redundant), 3 (relevant but need of minor revisions), and 4 (relevant and clear). The threshold for each item was set at equal or higher to 3 for both of the judges. As a result, 15 items were deleted. The remaining items showed an inter-judge reliability of 0.8.

The final questionnaire consisted of 20 self-administered items each rated on a 4-point Likert scale (0 = never to 3 = always). Specifically, seven items were hypothesized to address the first dimension, nine the second, and four the third. The global score was obtained by adding each item and ranged from 0 to 60, where higher scores indicated higher levels of TS. No reverse items were established.

### 2.4. Statistical analyses

Data were analyzed using IBM^®^ SPSS^®^ Statistics v.27, while the open-source statistical software JASP was used for the confirmatory factor analysis (CFA). The comparison between the three age groups on the nominal variable (gender) was made using the chi-squared test. Normality was tested through the skewness and kurtosis method. The Cronbach’s alpha and McDonald’s omega tests (>0.80) were performed with inter-item correlation to test questionnaire reliability; items with an over-threshold correlation can result in redundancy and multicollinearity, therefore items with high correlation (>0.70) were removed. Keiser-Meyer-Olkin (KMO; >0.60) and Bartlett’s sphericity tests (*p* < 0.05) were used to evaluate the adequacy and suitability of the sample before performing the factor analysis. Exploratory factor analysis (EFA) employed principal components analysis with oblique rotation (oblimin) was performed and enforced a four-factor solution to test the theoretical structure of WRT-Q. We adopted the oblimin or oblique rotation because it is more appropriate when the items of the questionnaire are supposed not to be orthogonal, that is, independent of each other, as in this case. To ascertain the factor solution CFA was performed. The goodness-of-fit of the model was based on: S-B Χ^2^/df *p* > 0.05, CFI > 0.90, IFI > 0.90, GFI > 0.90, SRMR < 0.08, and RMSEA between 0.05 and 0.08. The sample was randomly distributed in two subsamples (Group 1 *N* = 1,290 and Group 2 *N* = 1,296) to perform EFA and CFA, respectively. In the whole sample, an independent sample *t*-test was conducted to compare women and men subgroups on TS and one-way ANCOVA to compare TS between age classes with gender as a covariate. Finally, Spearman’s correlation analysis was performed to test the sample’s association of TS levels and continuous variable (age). The significance of all analyses was set at *p* < 0.05.

## 3. Results

Demographic data about the participants (*N* = 2,586) are illustrated in Table 1. The chi-squared test showed significant differences between age classes in gender distribution (*p* < 0.001), with a higher number of females than males in <35 years and 36–54 years classes and a lower number of females than males in the older subjects’ subgroup (>55 years).

**Table 1:** Socio-demographic characteristics of participants (*N* = 2,586).

	<35 years *N* = 271	36–54 years *N* = 1,696	>55 years *N* = 619		
	N (%)	N (%)	N (%)	*x* ^2^	*p*
Gender				24.125	**<0.001**
Women	144 (53.1)	911 (53.7)	262 (42.3)		
Men	127 (46.9)	785 (46.3)	357 (57.7)		

Significant values (*p* < 0.05) are highlighted in bold.

The demographic characteristics of the two subsamples are shown in Table 2. The chi-square test highlighted no significant differences in gender distribution; the independent sample *t*-test was also not statistically significant.

**Table 2:** Socio-demographic characteristics of subsamples.

	Group 1 *N* = 1,290	Group 2 *N* = 1,296		
	N (%)	N (%)	*x*^2^ (or *t*)	*p*
Gender			2.496	0.116
Women	637 (53.1)	680 (53.7)		
Men	653 (46.9)	616 (46.3)		
Age (years) M ± SD	47.29 ± 8.6	47.24 ± 8.7	0.156	0.708

M, Mean; SD, Standard Deviation.

### 3.1. Reliability

Before conducting reliability analyses, the data were checked for normality through the skewness and kurtosis method. The data were normal (skewness *=* 0.840, kurtosis = 0.789). Moreover, the variance was 0.570.

The questionnaire was found to be highly reliable (Table 3).

**Table 3:** Reliability of the WRT-Q.

	Cronbach’s Alpha	McDonald’s Omega
20-items version	0.904	0.896
19-items version	0.910	0.910
17-items version	0.896	0.897

Internal consistency reliability evidenced items with a correlation above the acceptable threshold. However, the correlation between items 18 and 19 (*r* = 0.815) suggested they were redundant in content (Supplementary Table S1). Thus item 19 was removed. Specifically, we kept item 18 as it emphasizes the compulsive behavior related to ICT use. Reliability analyses were performed a second time, and the questionnaire was highly reliable (Table 3).

### 3.2. Exploratory factor analysis

In the first sample (*N* = 1,290), the KMO value (0.943) and Bartlett’s sphericity test (*x*^2^ = 11309.647, *p* < 0.001) showed that the data were suitable for factor analysis. To explore the factorial structure of the WRT-Q, all 19 items left of the instrument underwent exploratory factor analysis (EFA) with oblique rotation (oblimin), which allows correlation between the latent factors. Out of 19 items, two items (i.e., 9 and 11) did not statistically match and did not reach the acceptable factor loading index (≥|0.40|) (Stevens, 1996). Thus the final version of the questionnaire was composed of 17 items and four factors, with a global score ranging from 0 to 51 and 62% of the variance explained by these four factors (Table 4).

**Table 4:** EFA with oblique rotation (oblimin) (*N* = 1,290).

	Factors			
	Quality of work-life	Intrusion	Cognitive overload	Psychophysical stress
Item 2	**0.852**	−0.106	0.016	0.027
Item 1	**0.846**	−0.025	−0.002	−0.062
Item 3	**0.789**	0.065	−0.103	0.155
Item 4	**0.645**	0.053	0.041	0.218
Item 5	**0.430**	0.268	0.356	−0.101
Item 18	−0.239	**0.760**	−0.049	0.086
Item 6	0.216	**0.739**	−0.024	−0.005
Item 17	0.222	**0.522**	0.076	0.197
Item 8	−0.058	−0.083	**0.841**	0.018
Item 16	−0.088	−0.029	**0.824**	−0.028
Item 14	0.102	−0.164	**0.526**	0.342
Item 7	0.172	0.383	**0.525**	−0.046
Item 10	0.095	0.160	**0.517**	0.077
Item 15	−0.022	0.073	−0.042	**0.814**
Item 12	−0.033	0.013	−0.022	**0.795**
Item 13	0.255	−0.012	0.187	**0.531**
Item 20	0.172	0.207	0.160	**0.502**
Item 11	0.290	−0.094	0.355	0.357
Item 9	0.380	0.324	0.229	0.073

EFA, Exploratory Factor Analysis; The items that reach the acceptable factor loading index (≥|0.40|) are highlighted in bold.

The factors resulted positively correlated; the factor correlations matrix of EFA is showed in Table 5.

**Table 5:** Correlation matrix of the factors in the EFA model.

	F1	F2	F3	F4
F1	1			
F2	0.285	1		
F3	0.504	0.208	1	
F4	0.48	0.294	0.411	1

F1, Quality of work life; F2, Intrusion; F3, Cognitive overload; F4, Psychophysical stress.

**Figure 1:**
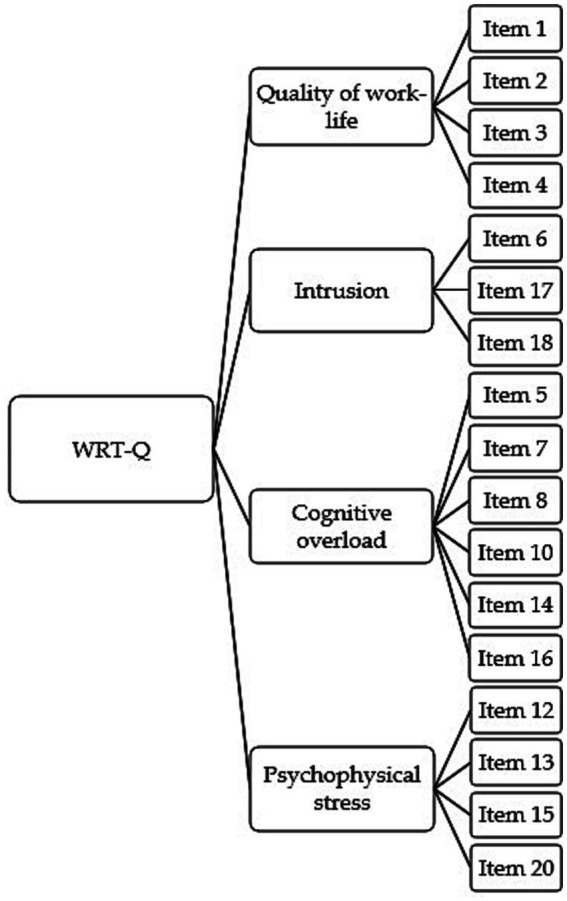
The path diagram of the four-factor model.

Based on the items’ contents, factors were renamed respectively: Quality of work-life (i.e., the negative effect of TS on concentration, performance, and well-being in the workplace context); Intrusion (i.e., hyper-connection and overlapping of private and working life due to ICT use); Cognitive overload (i.e., fatigue and exhaustion of one’s cognitive resources during work activities due to technology); Psychophysical stress (i.e., signs of stress at physical, emotional and mood level) (Figure 1). The Italian version of the WRT-Q is reported in Appendix A.

### 3.3. Confirmatory factor analysis

In the second sample (*N* = 1,296) the four-factor solution of WRT-Q was re-examined using CFA to determine its model fit. The Chi-square goodness of fit was not statistically significant (*X*^2^ = 443,211 df = 10, *p* < 0.001). The *X*^2^ is likely to be affected by a large population to be statistically significant; therefore, for the model of the WRT-Q, other multiple indices were used to judge the overall goodness of fit: CFI = 0.985; IFI = 0.985; GFI = 0.988; RMSEA = 0.071; and SRMR = 0.062. All the indexes were within acceptable ranges, which means that the four factors obtained from EFA were validated and the WRT-Q had a high goodness of fit. There was a positive correlation between the factors, with estimates ranging from *r* = 0.620 to *r* = 0.820, and there was a significant relationship among the factors (*p* < 0.01) (Table 6).

**Table 6:** Correlation matrix of the factors in the CFA model.

	F1	F2	F3	F4
F1	1			
F2	**0.690**	1		
F3	**0.700**	**0.620**	1	
F4	**0.820**	**0.700**	**0.810**	1

Significant values (*p* < 0.01) are highlighted in bold. F1, Quality of work life; F2, Intrusion; F3, Cognitive overload; F4, Psychophysical stress.

### 3.4. TS differences among age classes and gender subgroups

One-way ANCOVA showed that TS levels were significantly different in the three groups (*F*_2,2582_ = 9.597; *p* < 0.001) while controlling for gender. Bonferroni’s *post hoc* highlighted that there was a statistically significant difference between the 35 and 36–54 years old groups (*p* = 0.044) and between the 35 and over 55 years old groups (*p* < 0.001), stressing higher levels in older classes. Moreover, a statistically significant difference was highlighted between the 36–54 and over 55 age groups (*p* = 0.006), emphasizing a higher level of TS in older subjects (Table 7).

**Table 7:** Age-classes comparisons on TS levels.

Variable	<35 years *N* = 271	36–54 years *N* = 1,696	>55 years *N* = 619	*p*	<35 vs. 36–54		<35 vs. > 55		36–54 vs. > 55	
					Crit. diff.	*p*	Crit. diff.	*p*	Crit. diff.	*p*
WRT-Q total score M ± SD	11.45 ± 7.0	12.69 ± 7.6	13.76 ± 8.36	**<0.001**	−1.239	**0.044**	−2.376	**<0.001**	−1.136	**0.006**
WRT-Q range	Min 0/Max 35	Min 0/ Max 47	Min 0/ Max 48	–	–	–	–	–	–	–

Significant values (*p* < 0.05) are highlighted in bold. WRT-Q, Work-Related Technostress Questionnaire; M, Mean; SD, Standard deviation.

Independent-sample *t*-test showed no statistically significant difference between women and men on the TS level (*p* = 0.073) (Table 8).

**Table 8:** Comparison between women and men on TS levels.

Variable	Women *N* = 1,317	Men *N* = 1,269	*t*-test	*p*
WRT-Q total score M ± SD	13.03 ± 7.6	12.59 ± 8.0	1.421	0.073
WRT-Q range	Min 0/Max 47	Min 0/ Max 48	–	–

Significant values (*p* < 0.05) are highlighted in bold; WRT-Q, Work-Related Technostress Questionnaire; M, Mean; SD, Standard deviation.

In the whole sample, the TS level was positively correlated to age (*r* = 0.091, *p* < 0.001) (Table 9).

**Table 9:** The demographic correlates of TS levels.

Variable	*N* = 2,586	
	*r*	*p*
Age	0.091	**<0.001**

Significant values (*p* < 0.05) are highlighted in bold.

## 4. Discussion

The main aim of the current study was to validate a new, manageable Italian questionnaire to assess TS specifically specifically in an Italian banking context, which would overcome some of the limitations of previous inventories, i.e., questions concerning specific ICT, scarce or absent focus on behavioral TS manifestations, excessive length, and poor handling in the workplace. These aspects made previous tools scarcely efficient in occupational health surveillance routine, while our proposal is best oriented to be included in screening procedures in the banking workplace context. The WRT-Q was developed to increase the understanding of workers’ TS and to achieve this goal a literature review was conducted on work-related stress research and the evolution of the concept of TS, the last theoretical models, and the pre-existing psychometric tools. As a result, three key dimensions were initially conceived (causal attribution, stress and emotional reactions, and loss of control), and 20 items were generated to measure and describe the core point of each dimension identified on a 4-point Likert scale. The result of EFA and CFA showed 17 items and four factors, namely quality of work life, cognitive overload, intrusion, and psychophysical stress, thus supporting the multidimensional concept of TS (Tarafdar et al., 2007; Ragu-Nathan et al., 2008; Ayyagari et al., 2011; Salanova et al., 2013); moreover, the WRT-Q showed high internal consistency. The novelty of our tool lies in the attention paid to the adverse emotional effects of technostress regardless of the kind of job, role, and ICT adopted in everyday work life compared to the pre-existing inventories. Furthermore, more attention has been paid to the quality of work life instead of focusing on job satisfaction, unlike the other tools. This means a deep understanding of how TS negatively affects concentration, performance, and well-being in the workplace. Finally, an additional new element is represented by the dimension of cognitive overload as a consequence of prolonged use of ICT, highlighting how TS may impact an individual’s mental resources during work activities.

The second aim of this study was to compare subgroups (gender and age classes) on TS levels using the newly validated instrument.

About age, previous evidence reported that age and TS are negatively related, where younger individuals experience higher levels of digital stress (Ragu-Nathan et al., 2008; Hauk et al., 2019). On the other side, some studies also reported a positive relationship between age and TS, with higher levels in aging groups (Wang et al., 2008; Shu et al., 2011; Fuglseth and Sørebø, 2014). As expected, our research found a significant difference between the age groups, with a higher level of TS in the over-55 group. Moreover, differences between the under-35 and the 36–54 age groups highlighted how TS level increases proportionally with age, with a lower level in young subjects. As highlighted by previous research, the pace of technological change may contribute to higher job insecurity and lower self-esteem in older people than in younger determining increased levels of TS (Nimrod, 2018).

Regarding gender, differently from what hypothesized, no statistically significant difference was found; this result seems to highlight that women and men are equally prone to the TS phenomenon, differently from what emerged in previous studies (Ragu-Nathan et al., 2008; Tarafdar et al., 2011; Riedl et al., 2012; Marchiori et al., 2019; La Torre et al., 2020). Compared to our study, previous contributions are characterized by smaller samples (from 20 to 1,000 participants), younger people, imbalance distribution between women and men, and outdated tools to assess TS levels. These methodological differences may have contributed to different results; however further investigations are needed.

Before concluding, some strengths and limitations of our study need to be discussed. To the best of our knowledge, this is the first study aimed at developing a questionnaire to assess TS on such a large sample (Tarafdar et al., 2010; Nimrod, 2018; La Torre et al., 2020; Fischer et al., 2021). Furthermore, unlike previous tools, the WRT-Q represents a short, flexible, and comprehensive self-report questionnaire composed of only 17 items to assess TS multidimensionality.

However, some limitations must be highlighted. First, the study was targeted only at Italian bank employees, which represent a limited perimeter of application; however, the questions were conceived to assess TS as triggered by the use of any technological device, regardless of job role, BU, or occupation, so it is supposed to be suitable for different workplace contexts. Nonetheless, further studies are required to deepen the generalizability of results to other populations and work contexts. Second, no information about employees’ levels of burnout or other psychological illnesses was collected. Moreover, during the study procedure, all participants were on duty, thus we can reasonably exclude serious mental and general health issues. As a consequence, our data may not catch higher levels of stress, including technostress. Also, this point might explain why the median scores of technostress were fairly moderate. Third, in our study we did not include tools nor questionnaires assessing neighboring phenomena, thus we could not perform analyses of the convergent and/or discriminant validity. These additional points were beyond the aims of our study, although hopefully they can be the object of future research to deepen the validity of the WRT-Q. Fourth, we performed the analyses on two sub-groups drawn from the same populations, a condition that partially limits external variability conclusions. Future studies could deepen the generalizability of results comparing samples drawn from different populations, possibly performing also an invariance analysis.

## 5. Conclusion

This study contributed to current assessment of TS by developing a new Italian valid instrument for its evaluation in the banking context and in particular very efficient for health surveillance screening. Future research will contribute to validating the tool in in other work contexts. Moreover, further studies should focus on establishing further convergent and/or discriminant validity, both with other TS pre-existing tools and with other potentially related measures, particularly in occupational stress and attitude in ICT usage. Finally, in our sample average levels of TS were relatively low, although some individual scores were high. To deepen the role of factors able to buffer or jeopardize TS was beyond the scope of the study. However, additional evidence to cast light on the issue are required.

## Data availability statement

The raw data supporting the conclusions of this article will be made available by the authors, without undue reservation.

## Ethics statement

The studies involving humans were approved by Joint Ethical Committee for Research of Scuola Normale Superiore, Scuola Superiore Sant’Anna, and IMT School for Advanced Studies Lucca. The studies were conducted in accordance with the local legislation and institutional requirements. The participants provided their written informed consent to participate in this study.

## Author contributions

DP: Conceptualization, Data curation, Formal analysis, Investigation, Methodology, Software, Validation, Visualization, Writing – original draft. ER: Funding acquisition, Project administration, Resources, Supervision, Writing – review & editing. MO: Conceptualization, Data curation, Investigation, Methodology, Software, Validation, Visualization, Writing – review & editing.

## Funding

This work was supported by Intesa Sanpaolo Innovation Center S.p.A. The research was conducted under a cooperative agreement between IMT School for Advanced Studies Lucca, Intesa Sanpaolo Innovation Center S.p.A., and Intesa Sanpaolo S.p.A.

## Conflict of interest

The authors declare that the research was conducted in the absence of any commercial or financial relationships that could be construed as a potential conflict of interest.

## Publisher’s note

All claims expressed in this article are solely those of the authors and do not necessarily represent those of their affiliated organizations, or those of the publisher, the editors and the reviewers. Any product that may be evaluated in this article, or claim that may be made by its manufacturer, is not guaranteed or endorsed by the publisher.

## Appendix A

Work-Related Technostress Questionnaire

The WRT-Q is protected by copyright. All rights reserved.

Per favore, indica con quale frequenza solitamente ti capita di provare ciascuna delle sensazioni descritte mentre lavori.

1. L’utilizzo prolungato e/o simultaneo di più apparecchi tecnologici per lavorare riduce il mio livello di concentrazione e mi fa distrarre più facilmente
  Mai  Qualche  volta  Spesso  Sempre
2. L’utilizzo prolungato e/o simultaneo di più apparecchi tecnologici per lavorare influisce negativamente sul mio rendimento al lavoro
  Mai  Qualche volta  Spesso  Sempre
3. L’uso continuativo e/o simultaneo di più apparecchi tecnologici per lavorare influisce negativamente sulla mia qualità di vita lavorativa
  Mai  Qualche volta  Spesso  Sempre
4. L’utilizzo prolungato e/o simultaneo di più apparecchi tecnologici per lavorare è per me causa di ulteriore stress sul lavoro
  Mai  Qualche volta  Spesso  Sempre
5. A causa dell’aumentata complessità degli strumenti tecnologici, ho l’impressione di aver subito un incremento di carico lavorativo
  Mai  Qualche volta  Spesso  Sempre
6. A causa delle nuove tecnologie per lavorare, ho la sensazione di dover essere sempre reperibile per i colleghi
  Mai  Qualche volta  Spesso  Sempre
7. Per tenermi al passo con le nuove tecnologie e i continui aggiornamenti ho la sensazione di dover sacrificare molto più tempo per lavorare
  Mai  Qualche volta  Spesso  Sempre
8. La mancanza di conoscenze tecniche e metodologiche adeguate per l’utilizzo di apparecchi, software ecc. sul lavoro mi manda in crisi
  Mai  Qualche volta  Spesso  Sempre
9. I problemi tecnici che possono succedersi mentre lavoro, comportano perdite di tempo e continue interruzioni che mi stressano molto
  Mai  Qualche volta  Spesso  Sempre
10. L’utilizzo frequente di apparecchi tecnologici per lavorare mi causa dei disturbi fisici (emicrania, bruciore agli occhi, calo della vista ecc.)
  Mai  Qualche volta  Spesso  Sempre
11. Quando lavoro con apparecchi tecnologici mi sento più irritabile
  Mai  Qualche volta  Spesso  Sempre
12. Il dover utilizzare frequentemente apparecchi tecnologici mi fa sentire meno sicuro di me, mi fa venire più dubbi e/o mi rende più difficile prendere decisioni
  Mai  Qualche volta  Spesso  Sempre
13. L’uso frequente e prolungato di apparecchi tecnologici per lavorare mi causa insonnia e sonno disturbato
  Mai  Qualche volta  Spesso  Sempre
14. Quando lavoro con apparecchi e mezzi tecnologici, preferisco utilizzare procedure che conosco bene anche se più lente di altre nuove da imparare
  Mai  Qualche volta  Spesso  Sempre
15. Ho la sensazione che l’uso dei mezzi tecnologici sul lavoro invada troppo la mia vita
  Mai  Qualche volta  Spesso  Sempre
16. Quando ricevo avvisi di messaggi, mail ecc. per lavoro non posso fare a men di leggerli subito, anche al di fuori dell’orario di lavoro
  Mai  Qualche volta  Spesso  Sempre
17. Interagire frequentemente e a lungo con apparecchi tecnologici mi causa sensazioni di ansia e tensione
  Mai  Qualche volta  Spesso  Sempre
  Scoring
  Mai=0  Qualche volta=1  Spesso=2  Sempre=3.
