# Supplementary material for: A new scale to assess technostress levels in an Italian banking context: the Work-Related Technostress Questionnaire
Source: Front Psychol. 2023 Sep 1;14:1253960. doi: 10.3389/fpsyg.2023.1253960 (PMC10505406; doi:10.3389/fpsyg.2023.1253960)
Supplement: Supplementary file 1 [file Table_1.DOCX]

**Table S1.** *Inter-item correlation of the WRT-Q*

|  | IT 1 | IT 2 | IT 3 | IT 4 | IT 5 | IT 6 | IT 7 | IT 8 | IT 9 | IT 10 | IT 11 | IT 12 | IT 13 | IT 14 | IT 15 | IT 16 | IT 17 | IT 18 | IT 19 | IT 20 |
| --- | --- | --- | --- | --- | --- | --- | --- | --- | --- | --- | --- | --- | --- | --- | --- | --- | --- | --- | --- | --- |
| IT 1 | 1 |  |  |  |  |  |  |  |  |  |  |  |  |  |  |  |  |  |  |  |
| IT 2 | .666 | 1 |  |  |  |  |  |  |  |  |  |  |  |  |  |  |  |  |  |  |
| IT 3 | .561 | .633 | 1 |  |  |  |  |  |  |  |  |  |  |  |  |  |  |  |  |  |
| IT 4 | .515 | .572 | .661 | 1 |  |  |  |  |  |  |  |  |  |  |  |  |  |  |  |  |
| IT 5 | .418 | .436 | .481 | .509 | 1 |  |  |  |  |  |  |  |  |  |  |  |  |  |  |  |
| IT 6 | .303 | .280 | .364 | .354 | .371 | 1 |  |  |  |  |  |  |  |  |  |  |  |  |  |  |
| IT 7 | .381 | .393 | .412 | .449 | .578 | .455 | 1 |  |  |  |  |  |  |  |  |  |  |  |  |  |
| IT 8 | .314 | .338 | .288 | .360 | .395 | .140 | .461 | 1 |  |  |  |  |  |  |  |  |  |  |  |  |
| IT 9 | .456 | .451 | .511 | .509 | .468 | .452 | .476 | .374 | 1 |  |  |  |  |  |  |  |  |  |  |  |
| IT 10 | .331 | .352 | .366 | .405 | .363 | .274 | .407 | .427 | .471 | 1 |  |  |  |  |  |  |  |  |  |  |
| IT 11 | .429 | .510 | .513 | .52 | .457 | .243 | .443 | .439 | .424 | .408 | 1 |  |  |  |  |  |  |  |  |  |
| IT 12 | .300 | .295 | .374 | .377 | .299 | .285 | .338 | .278 | .36 | .318 | .344 | 1 |  |  |  |  |  |  |  |  |
| IT 13 | .425 | .473 | .491 | .550 | .437 | .286 | .429 | .373 | .436 | .416 | .560 | .439 | 1 |  |  |  |  |  |  |  |
| IT 14 | .370 | .449 | .381 | .434 | .40 | .166 | .413 | .497 | .354 | .354 | .566 | .311 | .510 | 1 |  |  |  |  |  |  |
| IT 15 | .308 | .342 | .374 | .415 | .317 | .291 | .355 | .231 | .346 | .278 | .384 | .468 | .437 | .346 | 1 |  |  |  |  |  |
| IT 16 | .297 | .307 | .271 | .306 | .355 | .149 | .368 | .476 | .325 | .344 | .380 | .209 | .346 | .411 | .202 | 1 |  |  |  |  |
| IT 17 | .372 | .388 | .471 | .435 | .431 | .501 | .469 | .231 | .453 | .308 | .419 | .317 | .43 | .323 | .399 | .276 | 1 |  |  |  |
| IT 18 | .078 | .052 | .098 | .104 | .078 | .333 | .135 | -.043 | .121 | .093 | .038 | .077 | .076 | .012 | .155 | .025 | .232 | 1 |  |  |
| IT 19 | .069 | .062 | .107 | .097 | .094 | .352 | .123 | -.073 | .116 | .065 | .026 | .054 | .050 | -.012 | .148 | -.004 | .251 | **.815** | 1 |  |
| IT 20 | .422 | .467 | .507 | .533 | .438 | .363 | .457 | .357 | .492 | .397 | .501 | .416 | .581 | .474 | .493 | .328 | .491 | .224 | .214 | 1 |

IT=Item; The over-threshold inter-item correlations are highlighted in bold
